# Specifics of anticipatory competence of adolescents with speech pathology

**DOI:** 10.1192/j.eurpsy.2024.1367

**Published:** 2024-08-27

**Authors:** A. Akhmetzyanova, T. Artemyeva

**Affiliations:** Department of Psychology and Pedagogy of Special Education, Kazan Federal University, Kazan, Russian Federation

## Abstract

**Introduction:**

Adolescents with speech pathology experience disturbances in sound pronunciation, phonemic processes, poor vocabulary, insufficiently formed grammatical structure, and disturbances in coherent speech. The specifics of the emotional-volitional sphere of adolescents in this group are anxiety, isolation and negativism prevent the establishment of full social contacts with peers and adults and complicate the formation of their anticipatory competence.

**Objectives:**

Studying the specifics of the anticipatory competence of adolescents with speech pathology.

**Methods:**

The study involved 56 adolescent children aged 11-15, attending an educational institution for children with disabilities, diagnosed with general speech impairment level 2. The study was carried out using the following methods: “Achenbach Questionnaire”, “Test of Anticipatory Consistency” by V.D. Mendelevich, “Anticipation of the outcome of a situation with a violation of the norm” by V.P. Ulyanova and the author’s methodology “Studying the anticipatory competence of adolescents” by Akhmetzyanova A.I., Artemyeva T.V.

**Results:**

It was revealed that adolescents with speech pathology experience difficulties in mastering the material, it is difficult for them to concentrate their attention on the task and bring the work they have started to the end. Adolescents of this nosological group face difficulties in predicting the outcome of situations and the consequences of their own behavior in a situation of social interaction, find it difficult to control time in the process of doing homework and organizing leisure time, make a forecast of various situations that may arise at school and family, make new acquaintances, and communicate freely with parents, teachers and peers.

**Conclusions:**

The level of speech development influences the formation of personal-situational, speech-communicative anticipatory competence of adolescents, and the ability to predict speech situations. Teenagers with speech pathology need help from adults This paper has been supported by the Kazan Federal University Strategic Academic Leadership Program.

**Disclosure of Interest:**

None Declared

